# Resilience, vulnerability and adaptability: A qualitative study of COVID-19 lockdown experiences in two Henan villages, China

**DOI:** 10.1371/journal.pone.0247383

**Published:** 2021-02-25

**Authors:** Xiao Tan, Yao Song, Tianyang Liu

**Affiliations:** 1 Centre for Contemporary Chinese Studies, Asia Institute, The University of Melbourne, Melbourne, Victoria, Australia; 2 School of Humanities and Social Science, The Chinese University of Hong Kong (Shenzhen), Shenzhen, Guangdong, China; 3 School of Political Science and Public Administration, Wuhan University, Wuhan, Hubei, China; Institute for Advanced Sustainability Studies, GERMANY

## Abstract

**Background:**

The Chinese government’s early handling of COVID-19 has been perceived as aggressive and oppressive. Many of the most radical measures were adopted in Henan province, immediately north of Hubei, the pandemic’s epicentre in China. However, little is known about how rural residents—a group systematically disadvantaged in Chinese society—responded to authorities’ draconian restrictions.

**Methods:**

To understand the lockdown measures and rural community responses at the grassroots level, face-to-face interviewers were conducted with both village cadres and villagers from two Henan villages in May and June 2020. The interviews were analysed with qualitative content analysis methods, with the coding process guided by the concepts of resilience, vulnerability and adaptability from the literature on disaster risk reduction.

**Results:**

We found that the lockdown measures were indeed radical and disproportionate relative to the level of risk presented; however, they were largely accepted by villagers. This contradiction can be explained by two key contributing factors: (i) shared interests of individual villagers and the converged goal of government and civil society, and (ii) tacit flexibility in COVID-19 adaption strategies to tackle conflict resulting from goal diversion between citizens and local governments.

**Conclusions:**

These findings highlight the nuances of ground-level politics. Despite their ‘radical’ nature, the lockdown measures were not implemented as simple top-down coercion. Instead, they involved, importantly, the bottom-up, localised response of villagers, and they were negotiated and adapted according to local circumstances.

## Introduction

In the face of the unprecedented COVID-19 crisis, China has rolled out a series of strict restrictions since late January 2020. Transport links were cut and housing complexes had access sealed off. Further, the Chinese government blanketed the country with hundreds of thousands of ‘grid workers’ (uniformed volunteers and young Party members) to guard the gates of residential complexes, enforce quarantines and conduct temperature checks [1]. In China, the spread of COVID-19 began to plateau around mid-February, and, since late March, lockdown measures have been gradually relaxed [2].

Opinions have been polarised on the strict measures in China. News reports in Western media have criticised the coercive and oppressive nature of the Chinese government’s approach, especially before COVID-19 became a global pandemic [3,4]. Some of these criticisms have resonated within China, for instance Fang Fang’s *Wuhan Diary*, which implicitly complained that measures adopted by local governments lacked ‘humanitarian spirit’. Conversely, Chinese authorities have insisted that the aggressive lockdown strategy was necessary for the country to win the battle against COVID-19 [5,6]. China’s approach won praise from the World Health Organization in February 2020—though the credibility of the World Health Organization has been fiercely questioned [7–9]. As the rest of the world struggles to contain the virus, and the death toll grows rapidly, some have even claimed that China’s model is the *only* proven and successful model to halt the spread of the virus [10].

The ongoing debate demonstrates an intense interest in China’s fight against COVID-19. However, little is known about how individuals inside China, particularly rural residents, have reacted to the government’s handling of the pandemic. Rural residents in China are estimated to earn only one-third of their urban counterparts, forming one of the highest urban–rural income differentials by international standards [11]. Due to the migration of around 290 million rural labourers to urban areas in search of employment, most of those living in the villages today are older members of the population and, as such, among those most susceptible to the potentially serious effects of the virus [12]. China’s rural areas were a critical part of the nation-wide quarantine: more than 600 million villagers constituted a major group during the lockdown. For this reason, this study intends to address the gap in the literature identified above by documenting first-hand experiences of rural residents during the lockdown and their responses to the strict restrictions imposed on their daily lives.

This paper presents fieldwork conducted in two villages in the northern and southern parts of Henan province. Henan was chosen because it was the pioneer in launching the most draconian restrictions in rural areas among all the Chinese provinces [13–16]. Radical border control was common in Henan villages, with local residents barricading themselves, destroying gateways and blocking roads [13,15–17]. The excessively cautious handling of Henan is also reflected in the fact that, compared to other provinces, Henan was significantly slower to reopen its economy. In April, when the spread of the virus was effectively managed, Henan’s return to work rate was only 64 per cent for manufacturing firms, 73 per cent for offices and 60 per cent for shopping malls. In Hunan (a province bordering Hubei to the south, where the number of people infected per million was 14.8, higher than Henan’s level of 13.3), the return-to-work ratios were as high as 77 per cent, 77 per cent and 74 per cent respectively [2].

### Existing knowledge of China’s COVID-19 experiences

A growing number of studies have evaluated the effectiveness of the measures taken by the Chinese government to respond to the crisis. These studies offer some evidence that the drastic control measures adopted by China substantially mitigated the spread of COVID-19 and prevented more infections [7,18–20]. The preliminary results of one study show that Chinese citizens are largely satisfied with the government’s measures [21]. Others have criticised some measures as having no scientific basis and thus, ineffective [22], although this view has been opposed by some, who defended policymakers as having to make decisions without adequate data during the early stage of the outbreak [23]. In a similar vein to mainstream reports in Western media, critics have tended to equate certain preventive measures—such as massive lockdowns and wide surveillance—with human rights violations [20,24].

While these studies are valuable in generating lessons for other countries in their own fights against COVID-19, there are important gaps. First, existing studies have tended to discuss the implementation of policies from the perspective of authorities [13,17]. There is a lack of understanding from the perspective of the people to whom the measures were applied. Second, most of the coverage of the lockdowns has focused on urban China, with limited understanding of how the pandemic affected approximately 600–700 million rural residents, who are systematically disadvantaged in Chinese society [25]. Third, few studies have explored the enabling factors of various government measures in the Chinese context, which is essential to evaluating the extent to which China’s model can be replicated elsewhere. By addressing the question of whether restrictive measures were positively accepted by rural residents, using the cases of Henan’s villages, this paper intends to contribute to a better understanding of how the lockdown worked and was received in rural China.

### Resilience, vulnerability and adaptability

The COVID-19 pandemic has similarities with natural disasters, both of which significantly disrupt social and organisational practices and call for resilience in society [26,27]. In disaster literature, resilience refers to ‘the capacity of a social system (for example, an organisation, city, or society) to proactively adapt to and recover from disturbances that are perceived within the system to fall outside the range of normal and expected disturbances’ [28,29]. Resilience is also a learning process that links ‘a set of adaptive capacities to a positive trajectory of functioning and adaption after a disturbance’ [30]. Essentially, resilience is about increasing adaptability at system level. Vulnerability is often considered to be the polar opposite of resilience [31]. Existing vulnerability studies either highlight the importance of resilience-building through local social systems, or suggest that vulnerability is related to economic powerlessness and the creation of sustainable livelihoods in a crisis or post-disaster context [32–37]. Central to these is an effort to develop the adaptive capacity of a community that partly depends on improving lives and livelihoods, with the choices and decisions made to adapt to the situation subject to a range of individual motivations.

In the ongoing COVID-19 emergency, the process of obtaining civil acceptance of the lockdown policy is partly dependent on reproducing resilience as an all-encompassing and somehow inevitable logic. This study moves beyond the neoliberal context dominating existing disaster literature (which tends to focus on resilience-as-governmentality) to focus on health emergencies in a semi-authoritarian system. Through the lens of resilience, vulnerability and adaptability, this paper explores how well-received the COVID-19 lockdown measures were by villagers in China, and why. Three reciprocal dimensions have at least partly shaped the experience of civil compliance: reduced vulnerability and strengthened rural resilience (i.e., community adaptability to a crisis); convergent goals between government and civil society with negotiation of marginal divergence of interests [38]; and multiple localised considerations, such as kinship, economic structures and lifestyles. This study both explores a new practical phenomenon and uses theoretical reflection to explain the social mechanisms underlying the implementation of the COVID-19 lockdown.

### The vulnerability of rural China in the context of COVID-19

Vulnerability presents a shift from conceiving the pandemic as ‘an event caused by an external agent to a more sociologically oriented interpretation of disaster as a complex, socially (as well as politically, environmentally and economically) constructed process’ [39]. A vulnerability assessment can uncover the structural drivers and situational factors that make specific groups vulnerable. These groups can then be prioritised for remedial action and help [39]. When this concept of vulnerability is considered in the context of COVID-19, social and academic observation produces two competing and contradictory versions of rural vulnerability.

One view is that rural society is inherently less vulnerable to the threat of COVID-19 [40]. During both SARS and COVID-19, mega-cities in China were badly affected while most rural areas were largely untouched. This disproportionate impact seems to support an urban-rural divide, legitimising a popular public opinion claiming that ‘the city is prone to plague, the countryside is an immune paradise’ [40]. Although not supported by scientific and statistical evidence, people tend to attribute the spread of COVID-19 to a radical urbanisation process in China. The large and densely inhabited urban areas, with overcrowded spaces for work and entertainment, provide ideal conditions for the rapid spread of the virus. In contrast, rural areas have smaller populations, scattered houses and self-sustaining systems of life. These spatial, economic and social characteristics do not appear to be as conducive to the spread of the virus.

In contrast to the idea that rural areas are less likely to be affected, some studies suggest that China’s rural population has a greater structural vulnerability during this public health crisis. China’s market reforms in the late 1970s have led to immense but unequal economic growth. A wide range of areas and population groups have been marginalised, demonstrated by income gaps, unequal access to education, poor health resources, labour deprivation and environmental degradation [41,42]. The economic literature suggests that, compared to their urban counterparts, rural households have benefited less from economic development [43]. According to the World Bank, China has one of the greatest urban–rural income divides in the world [11], with poverty overwhelmingly concentrated in rural areas [44,45]. Rural residents also tend to be particularly vulnerable to natural disasters, with many villagers falling into poverty following a bad harvest or other type of disaster [46,47]. A survey conducted in February 2020 indicated that many rural people were particularly afraid of falling back into poverty due to COVID-19 [48]. From an economic perspective, the disruption of the COVID-19 lockdown to income-generating activities would be likely to have a disproportionate impact on the economic wellbeing of Chinese villagers.

In addition to unequal income levels between urban and rural areas, the Chinese health system also has a strong urban bias. Both physical and human resources are mostly concentrated in urban areas, with the best services usually found in the university hospitals that are located in large cities [49,50]. Despite the government’s efforts to improve equality in the past decade, the country is struggling with an ageing and declining rural health workforce [51–53]. A typical village often only has one or two ‘village doctors’ (originally called ‘barefoot doctors’) who have received short-term training and are responsible for only preliminary public health and medical services [49,50]. Due to the continuous migration of young people from rural to urban areas in search of employment, rural China also has a larger concentration of older people, who are at greater risk of more serious illness if they are infected with COVID-19.

Lower income levels and inadequate health provision in rural areas have therefore weakened the significant physical and social advantages that could have offset vulnerabilities and helped individuals, groups and local communities to deal with the consequences of COVID-19. Therefore, COVID-19 might be expected to have a disproportionate effect on rural communities, making them more vulnerable. The existing evidence, however, suggests that the spread of the virus was limited in rural areas [25]. This contradiction could partly be explained by the effective prevention of cases being imported from urban areas, but the story remains incomplete without a clear picture of how the lockdown worked in rural communities.

## Materials and methods

### Research sites

The two villages studied are villages Z and B. Located in the northern part of Henan, village Z has a permanent population of 1,662, and another 1,500 migrant workers who spend much of their time working in cities. Located in the southern part of Henan, village B is closer to China’s outbreak epicentre, Wuhan (which is 40 minutes away by high-speed rail). The population of B is 2,660. Similar to village Z, most young people in village B have migrated to urban areas in search of better job opportunities, leaving agricultural work to older adults.

The local economy of village Z relies mainly on winter wheat. On average, each household has farmland of three to four *mu* (one *mu* = 0.067 hectares). The income from each *mu* of land is around 1,000 yuan per year (excluding labour, water, pesticides and fertiliser costs). Some older villagers and those who have migrated to cities have subcontracted their land, receiving an annual payment of 400–800 yuan per *mu*. In 2016, the provincial government identified village Z as experiencing poverty. Given the national agenda to eradicate absolute poverty by 2020 [54], the village received considerable attention from county and prefectural leaders. In village B, the local economy depends on rice. Like village Z, each household possesses around three *mu* of land, with each *mu* having the potential to generate 600–800 yuan annually.

There are only three main streets within village Z, with most of the villagers residing by, or close to, these main streets. It was relatively easy to set up barriers, and patrolling on the main streets was highly effective. In contrast, village B has a much more complex and fragmented geographical structure. Villagers reside in residential clusters, and these clusters, separated by mountain roads and rivers, are loosely connected in a segmented and polycentric network.

In both villages Z and B, the lockdown started on 25 January 2020, the first day of Chinese New Year. Consistent with the literature and news reports [13,15–17], both villages adopted radical measures, including road blockages, mass lockdowns, surveillance, and forced quarantine of people returning from Hubei.

The lockdown was relaxed in early April. At this time transportation blocks were removed. Villagers were allowed to return to work in other regions after providing required documents (e.g., proof of employment, proof that employing companies were reopened and welcome letters from the residential committees in the cities of employment). In both villages, no case of infection has been confirmed since the outbreak of the pandemic, which seems common according to a recent large-scale study showing very low infection rates in rural China outside Hubei [25].

### Data collection

Data collection occurred through face-to-face in-depth interviews with 16 respondents in villages Z and B. The interviews took place in May and June 2020, when people from outside the village were permitted to enter. Ethics clearance was approved by the Wuhan University, China. Written informed consent was obtained from the participants.

In each village, we interviewed eight people, consisting of (i) two village cadres (working on the village committee) who implemented the lockdown in their villages; (ii) two villager-volunteers who worked closely with the village cadres to facilitate the implementation of lockdown measures; (iii) two villagers from relatively well-off households; and (iv) two villagers from relatively poor households. In this way, we were able to achieve important diversity with our small sample. Also, within each category, we interviewed two people to cross-check information.

The initial contact with residents was established with the help of two colleagues who were born and bred in the two study site villages. Both left their home villages in the 2000s for further study and work, but have maintained close ties with family and friends who are still living there. Using the selection criteria introduced above, they helped us to identify potential candidates and line up interviews. One major limitation of this selection approach is that the interviewees were not randomly selected from among the village population. However, the introduction provided by our key informants ensured essential trust that helped us conduct smooth and productive interviews with the villagers.

The background information of our interviewees is summarised in Table 1. All interviewees remained in their village for the period from late January to April 2020. Therefore, they had all experienced, and in some cases implemented the lockdown themselves.

**Table 1:** Sampling for in-depth interviews in Henan villages.

Code	Village	Category	Basic information
Z01	Z	Village cadre	Village committee member (female representative) in her 40s
Z02	Z	Village cadre	Former village head in his 70s
Z03	Z	Villager from a relatively well-off household	Migrant worker in her 40s, who returned home for the Chinese New Year
Z04	Z	Villager-volunteer	Truck driver in his 40s, who volunteered his truck for food delivery during lockdown
Z05	Z	Villager from a relatively poor household	Wheat farmer in her 50s
Z06	Z	Villager-volunteer	Owner of one village grocery store in his 30s, who was actively engaged in organising food delivery during the lockdown
Z07	Z	Villager from a relatively well-off household	Pig farmer in his 40s
Z08	Z	Villager from a relatively poor household	Retiree in her 60s, who lives with her husband
B01	B	Villager from a relatively well-off household	Rice farmer in his 40s, who lives with his wife (their two sons are both migrant workers)
B02	B	Villager from a relatively poor household	Retiree in her 60s, who lives with her grandchildren
B03	B	Village cadre	Village head in his 40s
B04	B	Villager-volunteer	Rice farmer in his 40s
B05	B	Village cadre	Village committee member (accountant) in his 50s
B06	B	Villager from a relatively well-off household	Migrant worker in her 30s, who returned home for the Chinese New Year
B07	B	Villager from a relatively poor household	Freelancer in his 60s
B08	B	Villager-volunteer	Retiree in his 50s

The interviews were semi-structured, covering (i) how the lockdown was implemented in their village; (ii) the social and economic implications of lockdown for villagers’ lives; (iii) the reaction of villagers to the lockdown; and (iv) personal reflections. For each dimension, we prepared two to three questions in advance to ensure that the researcher provided each interviewee the opportunity to discuss and comment relatively comprehensively on their lockdown experience. All interviews were conducted in Mandarin, by a researcher trained in qualitative data collection methods. During the interview, participants’ responses guided the interviewer in deciding when and how to probe the emergent themes.

Participants were interviewed in their workplaces or homes, often with children and other family members present. We chose not to insist on conducting the interviews completely privately because, given the local culture, asking the respondent’s family members to leave the room for a private interview setup would bring unnecessary stress and make the respondent more likely to hold back during the interviews. However, we did not allow any non-family members to be present during the interviews. The main consideration was that the presence of non-family members can potentially distort how people responded to the questions. For example, the presence of implementers of lockdown would potentially result in the villager saying only the positive part of the lockdown.

Each interview lasted approximately one hour. The interviews were audio-recorded with the permission of participants, transcribed verbatim and de-identified. All the interviewees were informed that the interviews would be anonymous.

### Data analysis

We adopted a qualitative content analysis to analyse the interview content for this study [55,56]. The key concepts of resilience, vulnerability and adaptability guided the coding of the interview data, providing insights into the process of disaster risk reduction [26,37,57,58]. The core objective during the coding process was to ensure that all of the data relevant to each category was identified and examined [59,60]. Data coded under the same categories and subcategories were collated, compared and then summarised.

Two forms of triangulation were adopted to improve research validity. First, we intentionally selected interviewees in different roles and with different backgrounds. Importantly, we included four different categories of respondents in each village and two different people within each category. Such a diversity made it possible to triangulate data between interviewees. Second, researchers involved in this study worked as a team to analyse the data, which also provided a form of triangulation.

Another widely recognised dimension of analytic quality is generalisability. The danger is that researchers use exotic but untypical examples to make a general point [61]. To guard against this, we paid particular attention to always refer to specific interviews, comparing the information from our study with previous studies and relevant news reports on rural Henan. When needed, further information was given for readers to understand the context in which a point was made. In this way, we attempted to make the presentation of data as specific as possible to avoid over-generalisation.

## Results

### Lockdown implementation

In village Z, the lockdown was enforced by local governing bodies: the five members of the village committee formed a task force to carry out strict surveillance at the entrances of main streets. The two youngest members slept in a makeshift tent to ensure no undesired mobility. The three most respected people, members of the team of ‘five seniors’ (comprising a cadre, a teacher, a model worker, a specialist and a soldier), helped the village cadres guard the side streets. Additionally, two No. 1 secretaries, originally sent by the county government for poverty alleviation work, patrolled the main streets holding a horn loudspeaker. Whenever they saw people peering outside their yards, they would shout through their horn loudspeakers and ask them to return to their home immediately.

In village Z, a practice adopted to guarantee obedience to lockdown measures was a penalty method related to the ‘five constructions’ system (i.e., of the construction of the Party, stability and legality, a clean cadre team, rural civilisation, and a liveable environment). Under this system, each of the five dimensions was assigned ten points (thus, a total of 50 points), and the county government conducted quarterly inspections to assess how well villages performed in each dimension. The achievement of full points in each dimension would result in the older villagers (aged 65 years or above) receiving a bonus of 50 yuan per person per quarter. In the middle of the pandemic, the county authorities decided that should village officials be found incapable of executing lockdown measures, the village’s points would be deducted, and its senior villagers could not be rewarded accordingly.

In comparison, the lockdown was implemented less strictly in village B, partly due to the challenges presented by the fragmented geographical structure. The officials of village B guarded the main entrances, and hired people from those households experiencing poverty to help guard the checkpoints set up at the main entrances to village clusters. However, villagers were allowed to travel to the closest township to purchase food. Patrolling was done much less intensely.

Considerable effort was made to ensure that villagers would have access to food and other essentials during the lockdown. In village Z, there are two village grocery stores. Both remained open throughout the lockdown. The village committee set up a WeChat group for residents to make orders. Each store also established its own WeChat group to attract villagers. The village cadres worked closely with store owners to organise food and other essentials from the outside. They also convinced the store owners to make zero-contact deliveries to each household. When residents ordered some special commodity that the stores could not supply, the village committee would dispatch its members to arrange collective purchase once a day from the markets in the closest township. In village B, there is only one small grocery store in the main cluster, which was shuttered a couple of days after the lockdown. Consequently, the village committee permitted residents to purchase food and other essentials from the closest township by themselves. However, only one entry pass was assigned to each household, and they needed to present it every time they attempted to leave the village. In both villages, all interviewees suggested that there were few people who disobeyed the lockdown measures.

### Coping with the lockdown implications

In village Z the economic impact on agricultural production was minimal during the early stage of lockdown because there was no farm work in February. However, as the planting time for wheat started in early March, villagers became concerned with the potential economic loss caused by the lockdown and asked to work on their farms. Faced with strong demand, village cadres agreed that each household could dispatch one representative to attend to their farmland, with no more than 50 households permitted each day. Although the villagers had to wait for their turn, this policy appeared to be bearable. As one interviewee (intvw-Z05) suggested:

It was inconvenient, but still okay for us because wheat is fairly tolerant to drought. Also, we don’t have that much land so we can pretty much get work done.

However, since some villagers contracted large tracts of land or had land scattered in different places, it was difficult for them to complete their work in a day, and resulted in some tension between the affected villagers and the village cadres. In village B, since rice planting only started in April, when lockdown measures were relaxed, the villagers’ farming work was minimally affected.

As mentioned previously, village Z is identified as a village experiencing poverty. Those households experiencing poverty in village Z had long received a range of special treatment, such as pension and free food (intvw-Z04). Village officials even took the initiative to clean their homes for inspections from upper authorities (which is unsurprising given the bureaucratic institutions and strong pressure to complete poverty alleviation tasks in China [62]). In village B where there are fewer households experiencing poverty, to provide more assistance to these families, the village committees hired people from these households to help guard the checkpoints set up at the main entrances to village clusters. A salary of 500 yuan per month was offered (intvw-B07). This amount of money is very helpful, considering a local household could merely earn an annual income of between 1,800 and 4,000 yuan from agricultural activities on average. This move also proved beneficial for addressing the shortage of human resources faced by the village authorities, as there were neither ‘five seniors’ nor No. 1 secretaries in village B.

The progress in national health insurance reforms appeared to bring an additional sense of security, especially for those households experiencing poverty. As one interviewee (intvw-B05) elaborated:

Since the late 2016, those household experiencing poverty have been covered by an upgraded package of health insurance. If one household is categorised as experiencing poverty, they can be exempt from all kinds of medical payment. If they choose to claim from the Additional Serious Illness Insurance for the Impoverished, the provincial government will pay 60 per cent of the premium, and our county will shoulder the rest. If the disease is very critical, they can use the Catch-All Insurance of Serious Illness for the Impoverished, whose premium is split by [the authorities of] our city and county.

In terms of schooling, we found that the ‘homework gap’ [63]—where students cannot easily use the internet to access education—was limited in both villages. As in other urban and rural areas, the schools in the two villages were forced to shut down. Since the end of Chinese New Year in mid-February, the schools in both villages started to offer online classes while managing to ensure that students could keep up with study via a broadband connection. For instance, primary schools in village B asked teachers to record lectures and make them available online, while middle school teachers used Tencent Meeting, a cloud-based videoconferencing application, to deliver live teaching. Most of the families in both villages had a home desktop computer and high-speed internet connection, which echoes recent research suggesting that as many as 71 per cent of schools in rural China successfully ran online courses in February, and another 16 per cent started in March [25]. For children who had problems using wireless internet at home, schools in village B even promised to reimburse the cost of mobile data usage for their parents.

In both villages, many of the children are ‘left-behind’ by their migrant worker parents in the villages and typically reunite with their parents only once a year during the Chinese New Year. These ‘left-behind children’ are mostly taken care of by their grandparents. Despite all the inconvenience, the lockdown, which coincided with the Chinese New Year holidays, enabled some ‘left-behind children’ to gain a longer period of stay with their parents. As one parent of a six-year-old child (intvw-B06) expressed:

[Because of the lockdown,] I have some extra time to spend with my kid. My kid was always taken care of by her grandparents because I seldom return home. This time I am with her. She learned origami and helped with housework. She has grown up now. I also taught her how to do online shopping. Her grandparents have no idea how to do this.

### Villagers’ responses

All of our 16 respondents suggested that they observed few cases of lockdown rule violations and noted that the villagers were generally calm, and the village was relatively orderly. A few interviewees (intvw-Z01; intvw-Z02; intvw-B08) added that at the beginning, there was resistance from some villagers who found the lockdown to be inconvenient and were unwilling to follow the rules. However, the village committee members and frequently also their family members worked to persuade them of the reasons for lockdown (typically emphasising the dangers of the virus), resulting in those people changing their minds and becoming compliant.

In fact, for many, they held positive attitudes towards the lockdown from the beginning. As one interviewee (intvw-B02) expressed:

The atmosphere in the villages shifted immediately: the night before [the Chinese New Year], we were still hesitant whether to visit our relatives the next day, but by the early morning on the first day of the New Year, it became a new trend not to pay New Year’s visit, drop around or visit relatives.

Similarly, another interviewee (intvw-B07) observed that:

The villagers suddenly became even more nervous since they heard that there was a confirmed case in the neighbouring village. There was no one on the road those days. Some villagers even locked their doors with iron locks.

One interviewee (intvw-B01) recalled that he had heard a great deal of news from his well-informed son, who worked in Beijing and repeatedly warned him of the dangers of the virus, before the lockdown. This led the man to adopt many pre-emptive measures. As such, before village officials inspected his residence and instructed him what to do, he had already glued a warning on the tree outside his house, suggesting that he would refuse any visit including close relatives. As he recalled:

I was afraid of going out. Whenever my son left our yard and purchase food from outside, I reminded him of wearing a face mask, putting a plastic bag on head and wearing a raincoat. When he returned, I asked him to burn the plastic bag and put the raincoat out in the sun to disinfect.

In the village WeChat groups, villagers actively praised their leaders for their hard work (intvw-Z03; intvw-Z07). In village Z, two villagers volunteered to provide their vans for free use and act as drivers for collective purchases. The village committee insisted on compensating them, but the volunteers repeatedly refused any monetary benefit and were only willing to accept some symbolic compensation. In both villages, villagers also volunteered to send cash and gifts (bottled water, instant noodles, N95 masks and even private health insurance) to their village leaders to express gratitude.

Some other exemplar quotes include: ‘We appreciate what they [village cadres] did for us. They worked so hard, day and night’ and ‘I think the government did the right thing’. As one interviewee (intvw-Z03) phrased it:

We have all consciously participated in the prevention of the epidemic and have actively fought against it… In particular, staying at home is not only for our own benefit, but also for the country’s benefit. We are confident that the Party and the government will lead us to win this battle.

The strong affirmation of the local lockdown measures was also reflected in another villager’s (intvw-B06) response:

My mother runs a small business on the street all year round. She used to be very busy from the fifth day of the Lunar New Year and was very reluctant to take any rest. However, this time, because of the pandemic, she was very patient at home, and often actively called friends and relatives, sent WeChat messages, to remind them to ‘just get through this period of time and all will be fine’. I told my mother about a popular saying on the internet called ‘make contributions to the country by staying at home’. She nodded and said it made sense.

Similarly, another interviewee (intvw-B08) expressed:

In the past, this [COVID-19] would be called a plague, and people would definitely not be able to visit friends and relatives. We can understand… After all, it’s for everyone’s benefit. Otherwise, if one person got infected, the entire village would be in trouble. It was wise to lock down the village!

## Discussion

In both villages, the measures taken were disproportionate to the risks presented—mobility was highly restricted though there were no cases of infection. However, consistent with the existing literature that has suggested that citizen satisfaction with government measures is very high in China [21] and that most rural residents are very compliant [17], we found that the restrictive measures in villages Z and B were largely accepted by local people. In view of the findings above, the rural acceptance of the lockdown policies has two key contributing factors: (i) shared interests of individual villagers and the converged goal of government and civil society, and (ii) tacit flexibility in COVID-19 adaption strategies to tackle conflict resulting from goal diversion between villagers and local governments. In the following analysis, we elaborate how each of them contributed to the legitimisation of lockdown policies in local communities.

### An alignment of interests between villagers and rural authorities

The first important aspect for understanding villagers’ positive attitudes towards the radical measures is that the interests of villagers and the village committee were aligned by a shared interest in minimising the potential dangers of COVID-19. Based on our interviews, there has been a consensus on the dangers of the virus and the necessity of lockdown between villagers and rural officials. Such a consensus enabled a collective action of virus prevention, paving the way for the proactive engagement of village committee with various activities to facilitate the lockdown.

Built on the consensus, village leaders made many arrangements to cushion the negative implications resulting from the lockdown in both villages. In village Z, where a very strict form of lockdown was undertaken, considerable effort was made to organise the delivery of food and other essentials for everyone in the village. In both villages, people experiencing poverty had already benefitted from the poverty alleviation campaign and comprehensive health insurance arrangements. They received further assistance during the lockdown through, for instance, being hired to guard village entrances. Similarly, the children, especially the ‘left-behind children’, were able to resume their education and be accompanied by their parents during the lockdown.

Importantly, the alignment of interests hinged on close networks between groups and social cohesiveness. In both villages, most residents (especially the older ones) are tightly bound to their communities, which are kin-based and clan-ruled [64,65]. In village Z, the village committee and group of five seniors were drawn from the powerful families in the village who had the Z family name, which made up three-quarters of the population. With the exception of the two No. 1 secretaries dispatched from the county, nearly all the village officials who were on duty during the lockdown were familiar to the villagers. This was also the case for village B. As one interviewee (intvw-Z04) related:

There are two main clans in our village. Farmers are all relatives, even though some are connected by tenuous family links. Village leaders are also part of a big family. Those who are in charge of our village must think of their own family members. So, when the COVID-19 occurred, just like any previous great plagues in our village, how could they turn a blind eye and do nothing?

In village Z, the group of ‘five seniors’ hold a high level of authority and are greatly respected by local people. During the lockdown period, they repeatedly visited people who were unwilling to follow the rules. They explained the dangers of the virus and how essential the lockdown was, not only for the village but also for the entire country. This proved very effective; the resistance quickly waned (intvw-Z02).

In addition to the village officials and the ‘five seniors’, many people played a dual role and were policy enforcers as well as receivers. In normal times they had other occupations (their original professions are listed in Table 1), but during the pandemic they volunteered to help with enforcing the rules (in our study: intvw-Z04, intvw-Z06, intvw-B04, intvw-B08). As one villager-volunteer (intvw-B04) stated:

My family and I have been in the village for our entire lives so of course I want the village to be good… The higher levels [of the Chinese government] put much emphasis on rural areas this time and [the lockdown measures] are for the good of the village [so that I volunteered].

The use of customary relations facilitated cohesiveness among the officials and had a stabilising effect on the relationship between them and the villagers. The participation of villager-volunteers further blurred the line between village officials and villagers. This at least partly explains why, in the early days of controlling the pandemic, local government instructions were largely endorsed by the villagers. These psychological and social conditions strengthened community resilience during the pandemic by increasing the likelihood of effective responses to the risks, traumas and vulnerabilities associated with the lockdown.

The alignment of interests that underpins the community resilience and facilitates the civic acceptance of lockdown was an outcome of both individualistic, local determination and an imposed, top-down structure. The use of penalties related to the ‘five constructions’ system in village Z illustrates this point. Since almost every household had at least one older person, this mechanism tightly joined individual households’ interests with local policy priorities. Collective punishment quickly became a tool for policy implementation. When inspecting village Z during the lockdown, the county’s team noticed a villager walking on a street without any interference. They subsequently deducted all ten points for the ‘construction of Party branch’ dimension under the ‘five constructions’ system. The result was that each elder lost 50 yuan for that quarter. Disappointed by the loss, the seniors and their relatives turned to condemn the person who had violated the rule. In turn, this further enhanced the acceptance of the lockdown policies.

### Flexibility in lockdown implementation and room for negotiation

The ‘ruthless’ lockdown measures also derived their legitimacy from ambiguous processes of policy implementation characterised by compromise, contradiction and adjustment. This flexibility of the authoritarian Chinese system is not new; it has long been a focal point of interpretation by Chinese scholars. For example, Chinese governance often includes political devices or mechanisms that resemble Western liberal practices (e.g., limited, symbolic elections and institutional consultation). Considerable local autonomy to promote economic growth, system stability and fairness of the regime also exists [66–71]. Policymaking in China has long been described as ‘bargained’, ‘fragmented’ and ‘adaptive’ [72–75]. Sebastian Heilmann further considered it to have a ‘guerrilla’ character, which ‘allows constant adaptation to changes in the surrounding environment and justifies continual adjustments during implementation’ [76]. Our findings further these arguments by establishing the point that, even in the most securitised environment during the prevention of COVID-19, heavy-handed surveillance and policing were not experienced as a blanket but as a net, where holes were left on purpose for people to let off steam.

To begin with, local governments adapted the form of lockdown according to local demographics, including geographic features and human resources. Notably, a much more relaxed form of lockdown was chosen in village B, where the geographical structure was complex and fragmented and there was a lack of personnel to implement lockdown and organise food and deliveries. This use of flexibility could also be found in the decision to allow farming to continue in village Z from March 2020. As a province relying relatively heavily on agriculture, the Henan government was very concerned about the impact on agricultural production [77–79]. When the lockdown started to threaten production levels, the village leaders quickly adjusted their strategy.

Although the lockdown was strictly enforced, the government adopted a relatively lenient approach to those who failed to observe the lockdown rules. In the face of the initial resistance, the strategy was to persuade people to comply by emphasising the dangers of the virus. As one village official (intvw-Z01) stated:

Only very few people attempted to violate the lockdown rules. When they attempted to leave the village, [we] just convinced them. People all understand [the dangers of the virus]… Our village does not have any specific measure to punish the violators. If it exists, that is to convince them [to return home].

In village Z, some people attempted to water the vegetable patch close to their home. However, due to the strict form of lockdown measures chosen, this was considered to be against the rules. When such cases were identified, typically during the village officials’ patrol, they would call through their loudspeakers. People would return home immediately after being warned and no further action would be taken (intvw-Z01; intvw-Z07).

In village B, the main rule-breakers were some villagers who had secretly gathered to play mah-jong, a traditional game played with friends and relatives during the Spring Festival. In this case, village officials first attempted to warn the game-players and convince them to return home. After this persuasion proved fruitless, they called the police. However, the role of the police was only to frighten the players, with few coercive and punitive measures following (intvw-B03).

Essentially, the bargained (or fragmented and adaptive) forms of Chinese governance characterises ambiguity, unpredictability and uncertainty where a process of trial and error were at work. The practical sense of local officials may anticipate the situation where ill-adapted practices may incur negative sanctions, thereby creating incentives for them to produce the optimal response instantly. In this process, adapted and ill-adapted practices intertwine to sustain policy flexibility and the unceasing corrections of the policy outcome. In our cases, some people partially reserved from collective action. However, many of the civic reservations were eventually allowed by local authorities through local negotiation that aimed to sustain the adoptive and adaptive capacity of administrative resilience during the lockdown.

Overall, when people have a convergent goal or justification to virus prevention policy, rural resilience is consolidated by intra-group networks and local kinship. Measures of special care were developed to cope with groups identified as particularly vulnerable to COVID-19 and the social consequences of lockdown policy in the local context. Further, when the goals of government and rural households diverged, negotiation, compromise and flexibility in policy implementation were allowed to ensure the adaptive capacity of both administrators and local people in coping with local tension during the lockdown. By forging local consensus and reducing the subversive potential of a rural population in a lockdown emergency, rural conformity to the radical measures to combat COVID-19 becomes a consequence of both top-down coercion and bottom-up, localised resonances.

## Conclusions

Drawing on the first-hand experiences of rural residents in China’s fight against COVID-19, this paper uncovers the localised processes that took place amid the complex social, economic and physical environment of the COVID-19 emergency. Through interviews with local people in two Henan villages, we find that the lockdown measures were indeed radical. The measures were shaped by geographical structures and human resources, but overall, they were disproportionate relative to the level of risk presented. However, these measures were largely accepted and even welcomed by villagers, in sharp contrast to the expectation that draconian measures would be met with widespread resistance.

Guided by the theoretical literature on resilience, vulnerability and adaptability, we argue that this contradiction can be explained by two key contributing factors. First, the interests of villagers and the village committee were aligned with a shared recognition of the dangers of COVID-19 and a consensus concerning the necessity of strict lockdown measures. The shared interests hinged on close intra-group networks and social cohesiveness, which blurred the line between policy implementers and receivers in the villages, and were in some cases strengthened through localised administrative tactics. Equally importantly, there was a significant amount of flexibility in lockdown implementation, which allowed room for negotiation. These findings highlight the nuances of ground-level politics and further the arguments in the Chinese politics literature that policymaking in China is ‘bargained’, ‘fragmented’, ‘adaptive’ and of ‘guerrilla’ style [72–76]. Despite their ‘radical’ nature, lockdown measures did not reflect a simple implementation of top-down coercion, but also involved important bottom-up, localised resonances of villagers, reflecting negotiation and adaption according to local circumstances.

These combinations of governance and adaption strengthen rural resilience. According to this view, resilience is ‘positive’: it can be exported to and inculcated within society to help it prepare for or withstand extreme events, and form a new, stable, equilibrium. However, resilience is also ‘negative’: it produces a ‘politically debased’ (self-securing for less of a threat to themselves and in being so not a threat to the governance capacities of their states [80]) form of subjectivity that secures neoliberal governmentality [81]. In this sense, the Chinese authoritarianist mode of coping with COVID-19 has adopted numerous resilience approaches that have long been dominated in part by neoliberal logic. By incorporating ‘minor literature’ of Chinese responses to COVID-19 [82], the study reflects a notion of resilience as an ongoing interaction between various actors and logics. Because of this interactionist nature, we further realise that the process of COVID-19 emergency governance and related resilience-building can be far more contingent and contestable in both its characteristics and effects. This is because the ‘attempts to secure through resilience may paradoxically create new instabilities such that efforts to protect society may also end up creating further insecurities’ [81]. Future studies may examine how the potential excess of China’s systemic reaction to COVID-19 may give rise to unexpected, unforeseen, and disruptive effects in post-pandemic society.

The study is not without limitations. Given our focus on two villages, the interviews may not have covered all important dimensions of lockdown experiences in Henan. For example, while the lockdown had limited implications on wheat and rice production in our sample villages, the news reports suggest that it had more significant impacts on chicken husbandry in other Henan villages [83,84]. Those who experienced more serious economic loss would possibly have different and potentially more negative views towards the lockdown. Further, there seems to be more discontent in urban society that has been more severely afflicted by both COVID-19 and the social consequences of the virus prevention policy in China, evidenced in their variegated forms of contentious politics [82]. However, the purpose of this study is not to provide a comprehensive account of lockdown experiences in China, but to move beyond a top-down, state view on how COVID-19 issues were handled. This study presents an analysis from the ground, informed by micropolitical exploration that reveals unique, situated politics of everyday experience that, under pressure of lockdown, became reconfigured according to individual, community and national circumstances.

Finally, we note that the impact of COVID-19 is still unfolding. Although the pandemic is largely under control in China, the slow economic recovery, compounded by a global economic recession, is disproportionately hurting the villagers. A recent study shows that compared to urban migrant workers, migrant workers from rural areas find it much harder to find work post-lockdown [85]. As such, it remains unclear whether villagers’ positive responses can be sustained long-term. Future research is needed to closely monitor this marginalised group’s experiences and responses to the government’s handling of new and critical economic and social challenges in the post-pandemic era.
